# Stable isotopic analysis of water utilization characteristics of four xerophytic shrubs in the Hobq Desert, Northern China

**DOI:** 10.3389/fpls.2023.1103789

**Published:** 2023-06-09

**Authors:** Wu Yongsheng, Liao Zilong, Yu Xiangqian, Yin Qiang, Wang Hui, Gao Li

**Affiliations:** ^1^ College of Geographical Science, Inner Mongolia Normal University, Hohhot, China; ^2^ Institute of Water Resources for Pastoral Area, China Institute of Water Resources and Hydropower Research, Hohhot, China; ^3^ School of Earth Sciences and Engineering, Hohai University, Nanjing, China; ^4^ Institute of Grassland Research, Chinese Academy of Agricultural Sciences, Hohhot, China

**Keywords:** stable isotope, water utilization, water source, xerophytic shrubs, Hobq desert

## Abstract

Quantitative identification of water utilization characteristics of xerophytic shrubs is an important prerequisite for the selection and optimization of a regional artificial sand-fixing vegetation system. In this study, a hydrogen (δD) stable isotope technique was used to study the changes in water use characteristics of four typical xerophytic shrubs, *Caragana korshinskii*, *Salix psammophila*, *Artemisia ordosica*, and *Sabina vulgaris* in the Hobq Desert under light (4.8 mm after 1 and 5 days) and heavy (22.4 mm after 1 and 8 days) rainfall events. Under light rainfall, *C. korshinskii* and *S. psammophila* primarily used the 80–140 cm layer of soil water (37–70%) and groundwater (13–29%), and the water use characteristics did not change significantly after the light rainfall event. However, the utilization ratio of *A. ordosica* to soil water in the 0–40 cm layer increased from less than 10% on the first day after rain to more than 97% on the fifth day after rain, whereas the utilization ratio of *S. vulgaris* to soil water in the 0–40 cm layer also increased from 43% to nearly 60%. Under heavy rainfall, *C. korshinskii* and *S. psammophila* still used the 60–140 cm layer (56–99%) and groundwater (~15%), while the main water utilization depth of *A. ordosica* and *S. vulgaris* expanded to 0–100 cm. Based on the above results, *C. korshinskii* and *S. psammophila* primarily use the soil moisture of the 80–140 cm layer and groundwater, while *A. ordosica* and *S. vulgaris* use the soil moisture of the 0–100 cm layer. Therefore, the co-existence of *A. ordosica* and *S. vulgaris* will increase the competition between artificial sand-fixing plants, while the combination of the two plants with *C. korshinskii* and *S. psammophila* will avoid competition between artificial sand-fixing plants to some extent. This study provides important guidance for regional vegetation construction and sustainable management of an artificial vegetation system.

## 1 Introduction

China is one of the countries that is most seriously affected by sandstorms (A Bulletin of Status Quo of Desertification and Sandification in China, 2015). Planting artificial vegetation has long been one of the most effective measures used to reduce the damage caused by windstorms and to improve the living environment in sandy areas (Li et al., 2013). The artificial vegetation construction has shown remarkable progress over the last 60 years and has effectively prevented further desertification and promoted local habitat restoration and has even achieved desertification reversal in the areas with relatively more available water (Wang et al., 2008). However, several problems have been observed in practice, including declines in groundwater and the death of sand-fixing vegetation in some regions due to neglect of the vegetation carrying capacity. These factors directly affect the survival of sand-fixing vegetation, even threatening the ecological security and sustainable development of northern China (Cao, 2008). This lack of understanding concerning the water requirements of sand-fixing plants is an important cause of such problems (Turnbull et al., 2012; Li et al., 2013). Therefore, an in-depth understanding of water use characteristics of sand-fixing shrubs is an important prerequisite for building a sustainable sand-fixing vegetation system.

Stable isotopes are powerful tools for detecting water movement along the soil-plant–atmosphere continuum (Dawson et al., 2002). Isotopes have been widely used in arid and semi-arid environments to assess plant water uptake patterns, water competition, and partitioning among plants (Huang and Zhang, 2015). For example, it was reported that the *Populus fremontii* and *Salix gooddingii* near perennial and ephemeral streams in western Arizona used groundwater throughout the entire growing season, regardless of the groundwater depth (Busch et al., 1992). In the Mu Us sandy land of Northern China, water utilization characteristics of different types of plants showed that *Sabina vulgaris* and *Salix matsudana* consumed relatively deep soil water as well as groundwater, whereas *Artemisia ordosica* used only shallow soil water (Ohte et al., 2003). Zhao et al. (2008) evaluated water sources of riparian forest plants in the extremely arid region along the lower reaches of the Hei River basin in China by a multi-source mass balance method; they found that *Populus euphratica* and *Tamarix ramosissim* obtained over 93% and 90%, respectively, of their water from groundwater, while *Sophora alopecuroides* obtained more than 97% of its water from the soil layer at a depth of about 80 cm. All of these findings indicate that different plants have different water absorption patterns in terms of water utilization.

When groundwater cannot be directly used by plants, precipitation is an important environmental factor that determines the structure and function of dryland ecosystems (Huxman et al., 2004). The amount and intensity of precipitation will affect the recovery rate of soil water and the utilization efficiency of desert plants on precipitation and then in turn affect the survival, composition, and structure of species (Sala and Lauenroth, 1982; Dodd et al., 1998; Li et al., 2010). It has been reported that light precipitation events can effectively restore surface soil water, thus benefiting shallow root plants, whereas greater precipitation is beneficial to the restoration of deep soil water and to the plants with deep roots (Sala & Lauenroth, 1982; Li et al., 2010). Even within the same plant community, different plant species show different water use strategies to avoid competition through seasonal changes in water use (Ehleringer & Dawson, 1992). During seasonal water shortages or when there is no precipitation for a long time, deep-rooted plants will endure or avoid drought by absorbing deep soil water or groundwater (Zencich et al., 2002; Chimner and Cooper, 2004). This indicates that there is a competitive relationship between plants using the same depth of water.

In addition, in arid and semiarid regions, plants with different life forms use water sources from dissimilar soil layers, depending in part on the depth and degree of overlap of their roots (Schlesinger and Ehleringer, 2001; Schenk and Jackson, 2002). The pulse of shallow soil water and the diversification with respect to water use play important roles in determining the local patterns of species coexistence in arid ecosystems (Schenk and Jackson, 2002). The response of water use characteristics of different life form plants to rainfall changes in the Ordos Plateau indicated that the perennial grass *Stipa bungeana* and the herb *C. komarovii* take advantage of shallow water sources derived from light (<10 mm) rain events; the shrub *A. ordosia* utilized deeper soil water recharged by large (>65 mm) rain events, while *Cynanchum komarovii* relied primarily on rain events of intermediate (10–20 mm) size. The rainwater utilization patterns of the three species would allow the coexistence of *S. bungeana* and *A. ordosia* or the coexistence of *A. ordosica* and *C. komarovii* in various successional seral stages following a disturbance (Cheng et al., 2006). These results show that different types of plants can coexist with other shrubs due to their different water utilization characteristics. Therefore, an in-depth understanding of the water use characteristics of sand-fixing plants in the growing season and their corresponding laws concerning precipitation change is a prerequisite for the combination and collocation of different types of artificial sand-fixing vegetation (Asbjornsen et al., 2007).

The Hobq Desert is located in the northern part of the Ordos Plateau; it is an important energy base in China as well as an important part of the northern ecological security barrier (Wang and Yang, 2018). With the implementation of ecological environment construction projects such as the Three North Shelterbelt and the Beijing Tianjin sandstorm source control, the vegetation coverage in the sand area has been significantly improved (Wang and Yang, 2018). Thus, degradation of artificial sand-fixing vegetation is very common, and this has produced widespread concern about sustainable stability of sand fixation vegetation systems (Cao, 2008). Although previous studies have addressed various components of tree water sources (Ohte et al., 2003; Cheng et al., 2006) in the Ordos plateau, quantitative data on the water use characteristics of typical sand-fixing plants are still insufficient, and the water use mechanisms of species are not yet fully understood, particularly the relevance of regional precipitation conditions. In fact, quantitative identification of the water use characteristics of sand-fixing plants is an important ecohydrological basis for the selection and optimization of sand fixation vegetation systems as well as being necessary to improve the quality and stability of degraded ecosystems. Under the background of strengthening the construction of the ecological security barrier in northern China, it is particularly necessary to identify the water use characteristics of sand-fixing plants and their coexistence mechanisms. To further clarify the above issues, in this study, we aimed to (1) evaluate the effects of different rainfall events on soil moisture; (2) identify potential water use sources of sand-fixing plants; (3) quantify water utilization ratios of such plants under wet (1 day after rainfall) and dry (5 or 8 days after rainfall) conditions. We hypothesized that the water use characteristics of sand-fixing plants would change with the number of days after rainfall.

## 2 Materials and methods

### 2.1 Study area

**Figure 1:**
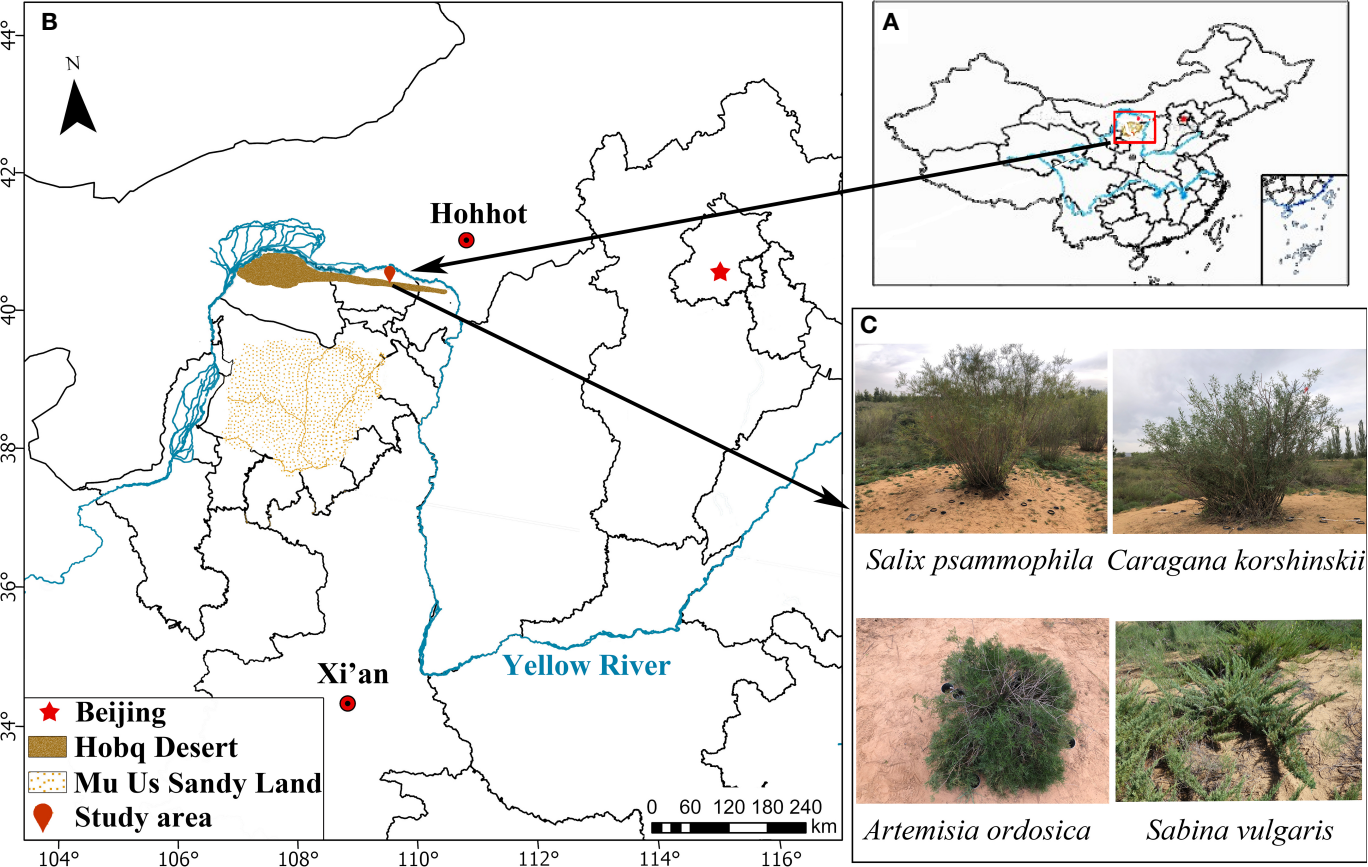
Schematic diagram of the geographical location of the study area **(A, B)** and the different types of sand-fixing shrubs **(C)**.

The study area was located in the eastern part of the Hobq Desert, belonging to Dalate Banner, Ordos City, Inner Mongolia Autonomous Region (**Figure 1**). The geographical location is 109° 00′–110° 45′ E, 40° 00′–40° 30′ N, and the altitude is about 1100 m. The region has a typical temperate continental climate. It is dry with little rain, cold in winter and hot in summer, with a large temperature difference between day and night. The annual average temperature is 6°C, and the lowest temperature is −32.3°C. The highest temperature is 38.3°C. The annual average precipitation is about 310 mm; the annual evaporation is 2600 mm, and the frost-free period is 156 days. Wind erosion and desertification in the study area are very severe. The soil comprises various aeolian sandy soils, and the vegetation type is dominated by *Artemisia ordosica.* As the constructive species in the region, *A. ordosica* grows on fixed and semi-fixed sandy land. The herbaceous plants include *Chenopodium acuminatum* and *Agriophyllum squarrosum*. The main nonnative plants include *Caragana korshinskii*, *Salix psammophila*, and *Sabina vulgaris* (Tang et al., 2018).

### 2.2 Methods

#### 2.2.1 Sample collection

In the study site, areas with small topographic differences and well-developed sand-fixing vegetation were selected to set up a fixed sample area. Sand fixing shrubs *Salix psammophila*, *Caragana korshinskii*, *Artemisia ordosica*, and *Sabina vulgaris* with average height and breast height diameter according to stand investigation were selected for isotope analysis. There were three individual xerophytic shrubs for each replication. To analyze the water utilization characteristics of different types of shrubs after rainfall events, this study selected the events of light rainfall (4.8 mm) on June 24 and heavy rainfall (22.4 mm) on August 18 and soil samples and plant samples on the first day after rainfall (June 25 and August 19) and 5–8 days after rainfall (June 30 for light rainfall and August 27 for heavy rainfall) for the comparison of plant water use characteristics after rainfall events. Groundwater samples were collected 1 day after rainfall (on June 25 and August 19). The maximum horizontal distance between the observation well used to collect groundwater and the plant sample plot was within 0.5 km.

Different types of sand-fixing plants were selected, and 2–3 sections of 3–4 cm branches were cut out from the middle part of shrubs and exposed to the sun; the bark and phloem of the branches were removed, and the remaining part was the wooden part. Plant samples were collected in the late morning hours (10 a.m.–12 p.m.) of each collection day and immediately placed into 12-mL sample bottles, sealed with Parafilm. Concurrent with plant tissue sampling, soil samples from each site were collected with a bucket auger at seven depths (0–20, 20–40, 40–60, 60–80, 80–100, 100–120, and 120–140 cm) from a borehole located beneath each of the three randomly selected mature sand-fixing shrubs. Soil samples were divided into two parts. Soil samples from the same type of shrub in the same layer were mixed for isotopic analysis, and the other part was used for gravimetric analysis of water content (*n* = 3). All the samples were stored on ice, and then transported to the laboratory for storage in a 4°C refrigerator.

#### 2.2.2 Sample analysis

Plant and soil samples were frozen and then thawed overnight using a cryogenic vacuum distillation method before water was extracted (Ehleringer and Osmond, 1989). The δD and ^18^O contents of the stem, soil, and ground water were measured using a Flash 2000 HT elemental analyzer (Thermo Scientific, Bremen, Germany) coupled to a Finnigan MAT253 isotope ratio mass spectrometer. The ^18^O content was determined by the H_2_O–CO_2_ equilibration method (Socki et al., 1999), and D content was determined by the gaseous H_2_–H_2_O equilibration technique (Coplen et al., 1991). Overall analytical precision of the spectrometer was ±< 0.2‰ for δ^18^O and ±< 1‰ for δD. The ^18^O and D contents of a water sample (δ sample) were expressed in delta notation (δ) relative to the Vienna Standard Mean Ocean Water (V-SMOW) standard:


$$\delta X=\left(\frac{R_{\text{samples }}}{R_{\text{SMOW }}}-1\right)\times 1000$$

where R represents the ratio of heavy to light isotopes (^18^O/^16^O or D/H). Hydrogen isotope data were used in this study.

#### 2.2.3 Data analysis

Plant water use from different soil depths was calculated using the Iso-Source mixing model (Phillips and Gregg, 2003). This model provides the distribution of proportions of feasible sources in the presence of a large number of potential sources and is based solely on isotopic mass balance constraints. All possible combinations of each source contribution (0–100%) were examined in 2% increments. Combinations that corresponded to the observed stable isotopic signatures of the mixture within a tolerance of 0.1 were considered feasible solutions; the frequency and range of potential source contributions were determined from these feasible solutions in accordance with the method described in detail by Phillips et al. (2005). We considered seven distinct water sources (20, 40, 60, 80, 100, 120, and 140 cm) and used δD data for model calculations. Graphical plotting was conducted with Origin 2018 software (OriginLab Corporation, Northampton, MA, USA).

## 3 Results

### 3.1 Changes in soil water content

**Figure 2:**
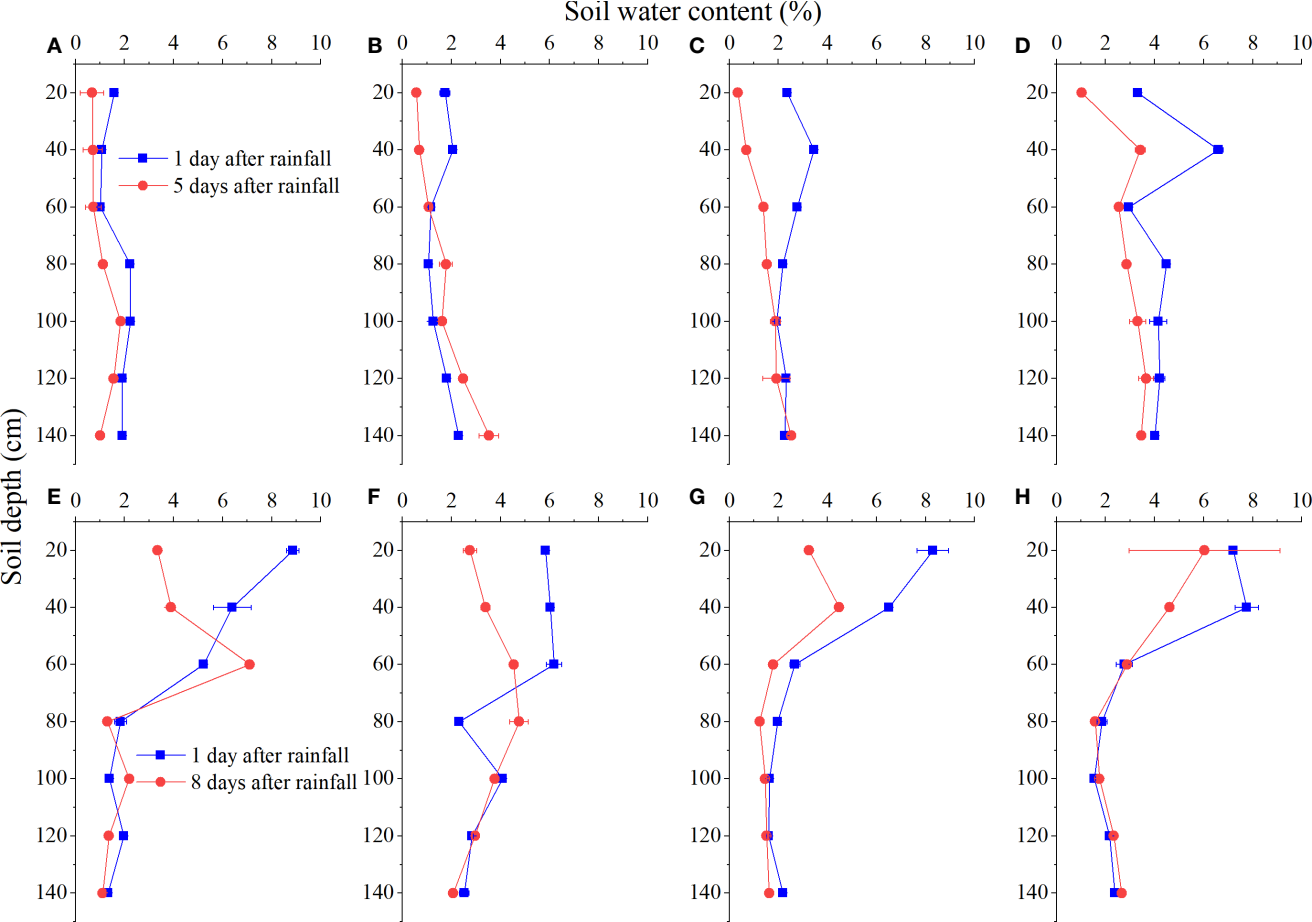
Response of soil water content in different types of sand-fixing vegetation to 4.8 mm **(A–D)** and 22.4 mm **(E–H)** rainfall. In the figures, A and E, B and F, C and G, and D and H represent the sand-fixing shrubs *C. korshinskii*, *S. psammophila*, *A. ordosica*, and *S. vulgaris*, respectively.

The changes in soil water content in different types of sand-fixing vegetation under light rainfall (4.8 mm) were very small. The soil water content of the 0–60 cm layer in different types of sand-fixing vegetation on the first day after rainfall was higher than that on the fifth day after rainfall, but their differences were limited within 2% (**Figures 2A–D**). After heavy rainfall (22.4 mm), the soil moisture content of the 0–60 cm layer in different types of sand-fixing vegetation on the first day after rainfall was higher than that on the eighth day after rainfall, with a difference of up to 6%, but the difference between 60–140 cm layers was still small (**Figures 2E–H**).

### 3.2 Variation in hydrogen isotope of xylem water and soil water and identification of water source

The changes in xylem water hydrogen isotopes in different species of sand-fixing plants after precipitation are shown in **Table 1**. On the first day after light rainfall, the order of hydrogen isotope values in the xylem of different types of sand-fixing plants was *S. psammophila* > *A. ordosica* = *S. vulgaris* > *C. korshinskii*. On the fifth day after rainfall, the order of hydrogen isotope values in the xylem of different types of sand-fixing plants was *A. ordosica* > *C. korshinskii* > *S. vulgaris* > *S. psammophila*. The hydrogen isotope contents in plant samples after rainfall were lower on the first day than on the fifth day after rainfall. For the heavy rainfall event, the order of hydrogen isotope values in the xylem of different species of sand-fixing plants on the first day after rainfall was *A. ordosica* > *C. korshinskii* > *S. psammophila* > *S. vulgaris*, while the order of hydrogen isotope values in the xylem of different types of sand-fixing plants on the eighth day after rainfall was *S. psammophila* > *C. korshinskii* > *A. ordosica* > *S. vulgaris*. The hydrogen isotope content in all plants decreased or was equivalent, except for that in *S. psammophila*, which increased.

**Table 1:** Changes in δD values in the xylem of different sand-fixing plants after light (4.8 mm) and heavy (22.4 mm) rainfall events (‰).

Rainfall (mm)	Treatment	Plants			
		*Caragana korshinskii*	*Salix psammophila*	*Artemisia ordosica*	*Sabina vulgaris*
4.8	1 day after rainfall	−53.75	−50.55	−58.21	−51.56
	5 days after rainfall	−58.48	−62.25	−67.35	−74.53
22.4	1 day after rainfall	−46.32	−59.04	−34.60	−74.07
	8 days after rainfall	−52.30	−50.59	−53.88	−72.17

**Figure 3:**
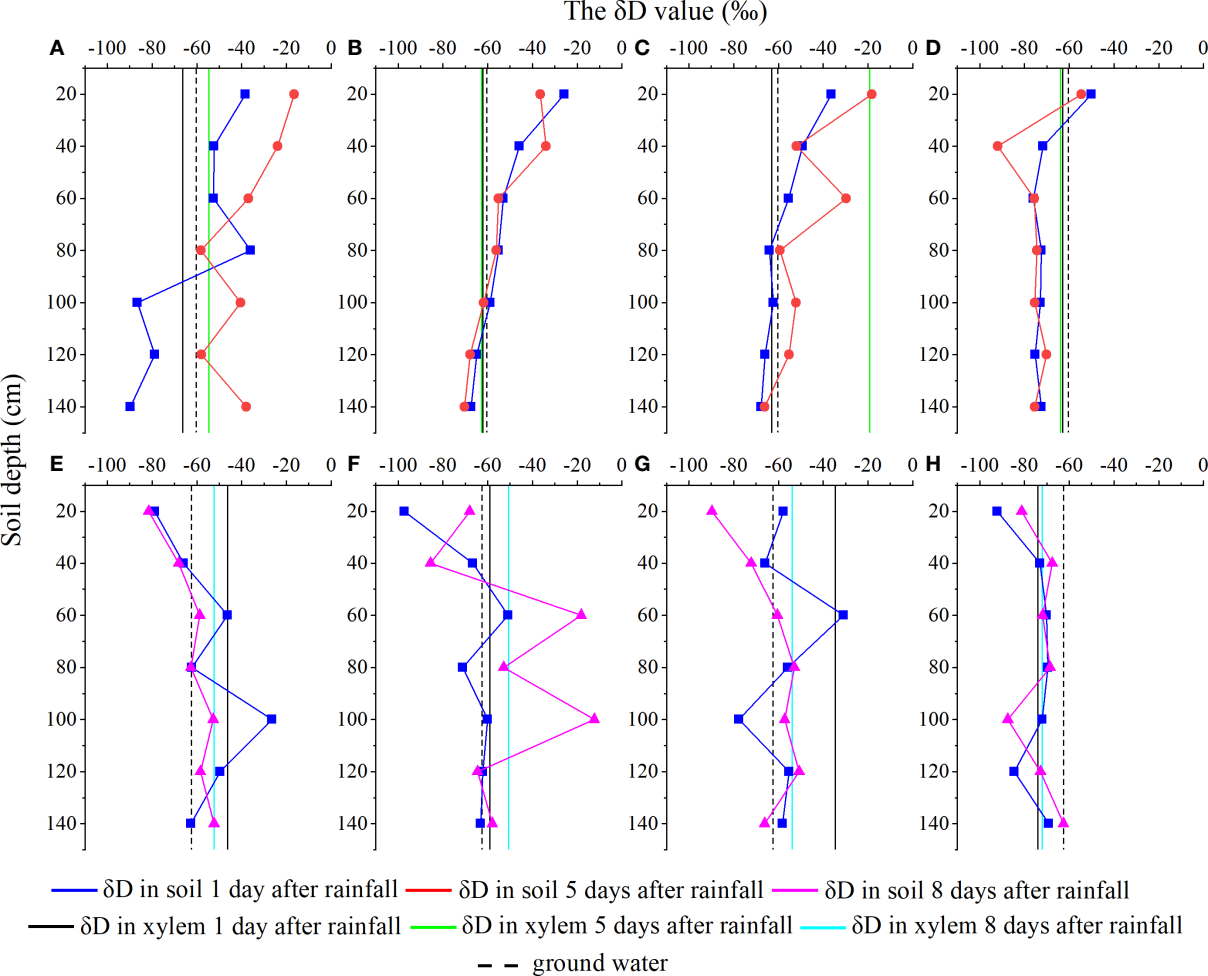
Changes in soil water hydrogen isotopes after small (p = 4.8 mm, **A–D**) and large (p = 22.4 mm, **E–H**) precipitation events. In the figures, a and e, b and f, c and g, and d and h represent the sand-fixing shrubs *C. korshinskii*, *S. psammophila*, *A. ordosica*, and *S. vulgaris*, respectively.

Under the condition of 4.8 mm precipitation, the changes in soil water isotope values on the first and fifth days after rainfall are shown in **Figure 3**. On the first day after rainfall, the soil water hydrogen isotope value of the *C. korshinskii* site in the 0–60 cm soil layer was greater than that on the fifth day, while in the soil layer below 80 cm, the soil water hydrogen isotope value on the fifth day after rainfall was greater than that on the first day after rainfall. The hydrogen isotope values of soil water on the first day after rainfall in the *S. psammophila* site were higher than those on the fifth day after rainfall and decreased with the increase of soil depth. In contrast to *A. ordosica*, in the 60 cm soil layer, the hydrogen isotope values of soil water in the *S. vulgaris* site on the first day after rainfall were higher than those on the fifth day after rainfall, whereas the hydrogen isotope values of soil water below the 60 cm soil layer tended to be stable (**Figures 3A–D**). Under the condition of 22.6 mm precipitation, the hydrogen isotope values of soil water in the *C. korshinskii* site on the first day after rainfall were higher than those on the eighth day after rainfall, and the hydrogen isotope values of soil water in the shallow layer (0–40 cm) were higher than those in the middle layer (40–80 cm) and deep layer (80–140 cm). On the first day after rainfall, the soil water hydrogen isotope value of the *S. psammophila* site increased after an initial decrease and then decreased with the increase of layer depth, and on the eighth day after rainfall, the level gradually decreased. The soil water hydrogen isotope values of *A. ordosica* and *S. vulgaris* site was very close, but the soil water hydrogen isotope values of the *A. ordosica* site showed a gradual decreasing trend with the increase of soil depth, while the soil water hydrogen isotope values of the *S. vulgaris* site showed a more gradual decreasing trend within the 0–80 cm soil layer and then tended to be stable (**Figure 3E–H**).

Water utilization sources of different types of sand-fixing plants changed after rainfall events (**Figure 3**). Under the condition of light rainfall (4.8 mm), *C. korshinskii* primarily used the 80–100 cm layer of soil water and groundwater on the first day after rainfall, and it used the 60–140 cm layer of soil water on the fifth day after rainfall, indicating that the water use characteristics of *C. korshinskii* did not change significantly after a light rainfall, and the plant still primarily used deep soil water (**Figure 3A**). *S. psammophila* primarily used the 100–120 cm soil water and groundwater on the first day after rainfall and the 100–120 cm soil water on the fifth day after rainfall (**Figure 3B**). *A. ordosica* primarily used the 100–120 cm soil water on the first day after rainfall and the 80 cm and 120–140 cm soil water on the fifth day after rainfall (**Figure 3C**). *S. vulgaris* primarily used soil water in the 20–40 cm layer on the first day after rainfall, and it still used soil water in the 20–40 cm layer on the fifth day after rainfall (**Figure 3D**).

Under heavy rainfall (22.4 mm), *C. korshinskii* primarily used soil water and groundwater in the 60 cm and 80–120 cm layers on the first day after rainfall, while it used soil water and groundwater in the 100 cm and 140 cm layers on the eighth day after rainfall, indicating that the water use characteristics of *C. korshinskii* changed significantly under heavy rainfall (**Figure 3E**). *S. psammophila* primarily used soil water and groundwater in the 100–120 cm layer on the first day after rainfall and that in the 40–120 cm layer on the eighth day after rainfall (**Figure 3F**). *A. ordosica* used soil water and groundwater in the 40–80 cm layer on the first day after rainfall and that in the 80–140 cm layer on the eighth day after rainfall. This shows that under heavy rainfall, the water use characteristics of *A. ordosica* changed from its use of middle (40–80 cm) soil water to its use of deep (80–140 cm) soil water (**Figure 3G**). *S. vulgaris* primarily used soil water in the 20–40 cm and 100–140 cm soil layers on the first and eighth days after rainfall, respectively, indicating that the water use characteristics of *S. vulgaris* had changed. *S. vulgaris* can use both deep and shallow soil water. No groundwater was used on the first day after rainfall, but groundwater was used on the eighth day after rainfall (**Figure 3H**).

### 3.3 Variation of water utilization percentages of different types of shrubs after rainfall

**Figure 4:**
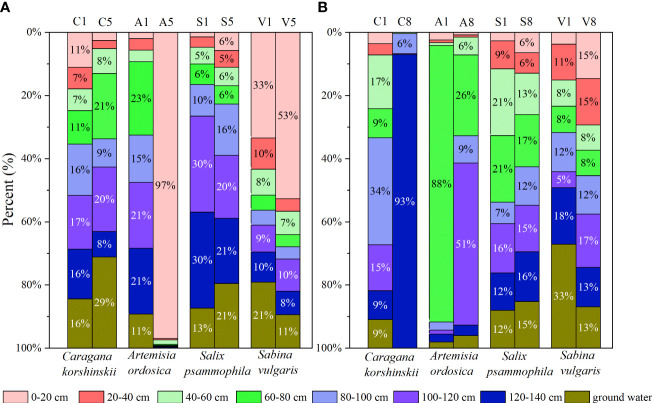
Variation in water use proportions in different species of sand-fixing plants after light (p=4.8 mm, **A, B**) and large (p=22.4 mm, **A, B**) rainfall events. In the figures, C1, A1, S1 and V1 and C5, A5, S5 and V5 represent the different types of sand-fixing shrubs after 1 and 5 days, respectively; C1, A1, S1 and V1 and C8, A8, S8 and V8 represent the different types of sand-fixing shrubs after 1 and 8 days, respectively.

The variation in water use proportions of different types of sand-fixing plants after precipitation is shown in **Figure 4**. Under the condition of light rainfall (4.8 mm), the water utilization source of *C. korshinskii* was relatively uniform on the first day after rainfall, with the proportion of water utilization in each soil layer ranging from 11% to 17%. On the fifth day after rainfall, *C. korshinskii* mainly used groundwater, with the utilization ratio as high as 29%, followed by its use of soil water in the 60–80 cm layer, with a utilization ratio of 21% (**Figure 4A**). *S. vulgaris* primarily used soil water in the 100–140 cm layer on the first day after rainfall, with a utilization ratio of 60%, while on the fifth day after rainfall, it used soil water and groundwater in the 100–140 cm layer, with a cumulative contribution of 62% (**Figure 4B**). The proportion of *A. ordosica* using soil water in the 60–80 cm layer on the first day after rainfall was 21%, while the proportion of *A. ordosica* using soil water in the 0–20 cm on the fifth day after rainfall had risen to 97% (**Figure 4A**). *S. vulgaris* used soil water in the 0–20 cm layer on the first day after rainfall, with a utilization rate of 33%, while on the fifth day after rainfall, it still primarily used soil water in the 0–20 cm layer, up to 53% **Figure 4A**).

Under the condition of heavy rainfall (22.4 mm), the proportion of sand-fixing plant *C. korshinskii* using soil water in the 80–100 cm layer on the first day after rainfall was 34%, and the proportion of sand-fixing plant *C. korshinskii* using soil water in the 120–140 cm layer on the eighth day after rainfall was 93% (**Figure 4A**). The cumulative contribution of sand-fixing plant *S. psammophila* to the use of soil water in the 40–80 cm layer on the first day after rainfall was 42%, while the proportions of soil water in each layer on the eighth day after rainfall were equivalent, ranging from 12% to 17% (**Figure 4A**). The proportion of sand-fixing plant *A. ordosica* using soil water in the 60–80 cm layer on the first day after rainfall was 88%, while the proportion of sand-fixing plant *A. ordosica* using soil water in the 100–120 cm layer on the eighth day after rainfall was 51% (**Figure 4A**). The proportion of sand-fixing plant *S. vulgaris* using groundwater on the first day after rainfall was 33%, while the proportion of sand-fixing plant *S. vulgaris* using water in each soil layer on the eighth day after rainfall was 12–17% (**Figure 4A**).

## 4 Discussion

### 4.1 Effects of precipitation on the soil moisture and hydrogen isotope composition in a sand-fixing vegetation restoration area

In arid and semi-arid sandy areas, water is a key factor affecting the structure, function, and stability of arid ecosystems and a central link connecting vegetation processes and hydrological processes (Sharma, 1991; Legates et al., 2011). Precipitation is the main source of soil water in arid and sandy areas when groundwater cannot be directly utilized. Therefore, the pattern and quantity of precipitation are crucial factors that determine plant colonization and growth (Dodd et al., 1998; Cheng et al., 2006). Soil water content is affected by rainfall infiltration supplementation, evaporation, and transpiration, and as such, it is in a constantly changing state (Li et al., 2010; Legates et al., 2011). It was reported that the hydrogen and oxygen isotope values in surface soil water are directly affected by precipitation due to the influence of precipitation infiltration δ (Lei et al., 2022). In this study, we found that in the light rainfall event, except for the *C. korshinskii* site sample plot, the changes of δD isotope at different sites were small, and the variation was concentrated in the surface layer. In the heavy rainfall event, soil water δD concentration of different vegetation sites became greater and showed a deepening trend (**Figure 3**). In this study, probably due to the combined effect of precipitation mixing and evaporation on shallow soil water, the amplitude of δD value changed significantly, and the magnitude of the change decreased with the increase of soil depth. The heavy rainfall had a clear replenishment effect on deep soil water, and the evaporation effect among shrubs was large (Wang et al., 2017). In addition, the architectures of different types of sand fixation plants are quite different, resulting in δD values showing clear differences.

### 4.2 Response of water use strategies of sand-fixing plants to rainfall changes

In arid sandy areas, light rainfall has a limited effect on soil moisture, and only rainfall greater than 10 mm can replenish deep soil moisture (Huxman et al., 2004; Turnbull et al., 2012; Li et al., 2013). Therefore, when the rainfall input is small, the change in surface soil water content is small, and the roots in the surface soil may be inactive under drought conditions (Flanagan et al., 1992). Plants can only rely on deep roots to absorb water from the soil (Ehleringer and Dawson, 1992; Ogle and Reynolds, 2004). When more precipitation is input, the amount of surface soil water recharged by precipitation will increase, and plants will increase the use of shallow water (Duan et al., 2008; Huang and Zhang, 2015). In June, when the rainfall is scarce and the soil moisture content is low, the surface soil moisture cannot be effectively supplemented by rainfall. In addition, in the early stages of the plant growth season, the plant has a high water demand and experiences strong evaporation, resulting in a significant increase in ground evapotranspiration and in the surface (0–40 cm) soil moisture content being lower than the deep soil moisture content (**Figure 2**). At this time, the deep root sand-fixing plants *C. korshinskii* and *S. psammophila* mainly use soil water in the 80–140 cm layer and directly use groundwater. The direct utilization ratio of the two sand-fixing plants of the deep soil water was greater than 60% (**Figure 4**), indicating that in the dry season, when the soil water content decreases, the sand-fixing plants *C. korshinskii* and *S. psammophila* can cope with the arid environment by increasing the utilization ratio of the deep soil water. Compared with *C. korshinskii* and *S. psammophila*, the roots of *A. ordosica* and *S. vulgaris* are concentrated in the range of 0–40 cm in the surface layer (Zhang et al., 2006; Wang et al., 2016). When the soil moisture content is low, their water use characteristics are still dominated by the use of the 0–20 cm layer soil moisture (**Figures 2**, **4**), and the utilization ratio of surface soil moisture was up to 97% in *A. ordosica* site, indicating that sand fixing plant *A. ordosica* has strong drought resistance and the ability to adapt to an arid environment. Influenced by the shade effect of plant aboveground architecture, the soil water content of sample *S. vulgaris* was generally higher than that of other sample plots, especially the surface soil water content (**Figure 2**). Sand-fixing plant *S. vulgaris* primarily used the 0–20 cm layer soil water but still has a considerable proportion (nearly 30%) of water from the deeper layer (80–140 cm) of soil water and groundwater, indicating that sand-fixing plant *S. vulgaris* can use both the shallow layer (0–40 cm) and deep layer (80–140 cm) soil water as well as its flexible water use characteristics and strong drought adaptability. In August, when the soil moisture conditions are better, the soil moisture content in the surface layer (0–40 cm) and middle layer (40–80 cm) is effectively supplemented by rainfall, resulting in a higher soil moisture content in the surface layer and middle layer than in the deep layer (**Figure 2**). At this time, sand-fixing plants *A. ordosica* and *S. vulgaris* will increase the utilization ratio of middle soil water. Compared with *A. ordosica* and *S. vulgaris*, although the shallow soil water of sand-fixing plants *S. psammophila* and *C. korshinskii* sample plots was effectively supplied by rainfall, *C. korshinskii* and *S. psammophila* still used the deep soil water to adjust their own water use strategies.

### 4.3 Significance of differences in water use characteristics of sand-fixing plants to regional vegetation restoration

On the whole, in June, when precipitation is scarce, sand-fixing plants *C. korshinskii* and *S. psammophila* primarily used soil water below 80 cm and ground water, while *A. ordosica* and *S. vulgaris* used soil water from the 0–40 cm layer. In August, when precipitation is relatively abundant, sand-fixing plants *A. ordosica* and *S. vulgaris* still used the soil water in the 0–40 cm layer, while sand-fixing plants *C. korshinskii* and *S. psammophila* used the soil water in the 80–140 cm layer. Under the light rainfall event, the water use characteristics of sand-fixing plants *C. korshinskii* and *S. psammophila* did not change significantly, indicating that the light rainfall event had little impact on the water use strategies of the plants, whereas the water use characteristics of sand-fixing plants *A. ordosica* and *S. vulgaris* were sensitive to light rainfall events. Under the condition of heavy rainfall, *C. korshinskii* and *S. psammophila* still used the deep soil water, while the water use depths of sand-fixing plants *A. ordosica* and *S. vulgaris* were expanded. To improve the utilization efficiency under limited water conditions in sandy areas, the mixed planting of sand-fixing plants *C. korshinskii* and *S. psammophila*, or *A. ordosica* and *S. vulgaris*, will increase the competition for water, thus causing the instability of sand-fixing vegetation systems. The mixed planting of sand-fixing plants *A. ordosica* and *S. vulgaris* with *C. korshinskii* and *S. psammophila* would alleviate the competition for water between sand-fixing plants to a certain extent, so that the soil water in each layer could be effectively used, thereby improving the stability of the windbreak and sand-fixing vegetation system. Our research results also found that except for limited utilization of groundwater by sand fixation plant *A. ordosica*, different species of sand fixing plants have different degrees of utilization of groundwater, and the maximum utilization of groundwater by sand fixing plant *S. vulgaris* can reach 33%, indicating that the large-scale planting of sand-fixing plants *C. korshinskii*, *S. psammophila*, and *S. vulgaris* may cause a decline in the groundwater level.

## 5 Conclusion

In this study, hydrogen isotopic composition of water pools (ground water, soil water, and xylem water) was determined and compared; possible water uptake sources of four typical xerophytic shrubs, *C. korshinskii*, *S. psammophila*, *A. ordosica*, and *S. vulgaris* in the Hobq Desert, China, were investigated. In general, under light rainfall, sand-fixing plants *C. korshinskii* and *S. psammophila* primarily used soil water in the 80–140 cm layer (37–70%) as well as groundwater (13–29%), and the water use characteristics were not changed significantly under the light rainfall event (4.8 mm), whereas the sand-fixing plants *A. ordosica* and *S. vulgaris* primarily used soil water in the 0–40 cm layer, and the utilization ratio significantly increased (up to 97%) at 5 days after rainfall. Under the condition of heavy rainfall, sand-fixing plants *C. korshinskii* and *S. psammophila* still mainly used soil water in the 60–140 cm layer (56–99%) as well as groundwater (~15%), while the main water utilization depth of sand-fixing plants *A. ordosica* and *S. vulgaris* expanded to 0–100 cm. Based on the above results, sand-fixing plants *C. korshinskii* and *S. psammophila* primarily used the soil moisture of the 80–120 cm layer and ground water, while *A. ordosica* and *S. vulgaris* used the soil moisture of the 0–100 cm layer and increased the utilization ratio of shallow soil water (0–40 cm) under the condition of light rainfall. Therefore, the co-existence of sand-fixing plants *A. ordosica* and *S. vulgaris* will increase the competition for water between artificial sand-fixing plants, while the combination of the two xerophytic shrubs *C. korshinskii* and *S. psammophila* will avoid the competition between artificial sand-fixing plants to some extent. During the deployment of artificial sand-fixing plants, the utilization of groundwater by *C. korshinskii*, *S. psammophila*, and *S. vulgaris* should be fully considered.

## Data availability statement

The original contributions presented in the study are included in the article/supplementary material. Further inquiries can be directed to the corresponding author.

## Author contributions

All authors listed have made a substantial, direct, and intellectual contribution to the work and approved it for publication.
